# Development of a reverse transcription-loop-mediated isothermal amplification (RT-LAMP) system for a highly sensitive detection of enterovirus in the stool samples of acute flaccid paralysis cases

**DOI:** 10.1186/1471-2334-9-208

**Published:** 2009-12-16

**Authors:** Minetaro Arita, Hua Ling, Dongmei Yan, Yorihiro Nishimura, Hiromu Yoshida, Takaji Wakita, Hiroyuki Shimizu

**Affiliations:** 1 Department of Virology II, National Institute of Infectious Diseases, 4-7-1 Gakuen, Musashimurayama-shi, Tokyo 208-0011, Japan; 2 Institute of Microbiology, Chongqing Center for Disease Control and Prevention, 8 Changjiang 2 Road, Yuzhong District, Chongqing 400042, PR China; 3 National Reference Laboratory of Poliomyelitis, Chinese Center for Disease Control and Prevention, Beijing, PR China

## Abstract

**Background:**

In the global eradication program for poliomyelitis, the laboratory diagnosis plays a critical role by isolating poliovirus (PV) from the stool samples of acute flaccid paralysis (AFP) cases. In this study, we developed a reverse transcription-loop-mediated isothermal amplification (RT-LAMP) system for a rapid and highly sensitive detection of enterovirus including PV to identify stool samples positive for enterovirus including PV.

**Methods:**

A primer set was designed for RT-LAMP to detect enterovirus preferably those with PV-like 5'NTRs of the viral genome. The sensitivity of RT-LAMP system was evaluated with prototype strains of enterovirus. Detection of enterovirus from stool extracts was examined by using RT-LAMP system.

**Results:**

We detected at least 400 copies of the viral genomes of PV(Sabin) strains within 90 min by RT-LAMP with the primer set. This RT-LAMP system showed a preference for *Human enterovirus species C *(HEV-C) strains including PV, but exhibited less sensitivity to the prototype strains of HEV-A and HEV-B (detection limits of 7,400 to 28,000 copies). Stool extracts, from which PV, HEV-C, or HEV-A was isolated in the cell culture system, were mostly positive by RT-LAMP method (positive rates of 15/16 (= 94%), 13/14 (= 93%), and 4/4 (= 100%), respectively). The positive rate of this RT-LAMP system for stool extracts from which HEV-B was isolated was lower than that of HEV-C (positive rate of 11/21 (= 52%)). In the stool samples, which were negative for enterovirus isolation by the cell culture system, we found that two samples were positive for RT-LAMP (positive rates of 2/38 (= 5.3%)). In these samples, enterovirus 96 was identified by sequence analysis utilizing a seminested PCR system.

**Conclusions:**

RT-LAMP system developed in this study showed a high sensitivity comparable to that of the cell culture system for the detection of PV, HEV-A, and HEV-C, but less sensitivity to HEV-B. This RT-LAMP system would be useful for the direct detection of enterovirus from the stool extracts.

## Background

In the global eradication program for poliomyelitis, the laboratory diagnosis plays a critical role by isolating poliovirus (PV) from the stool samples of acute flaccid paralysis (AFP) cases. The isolation procedure of PV have been established based on the cell culture system using a human rhabdomyosarcoma cell line (RD cells) and a mouse L cell line expressing PV receptor (L20B cells) [1,2]. The advantages of cell culture-based procedure are; 1) apparatuses for molecular diagnosis are not required, and 2) a high sensitivity (detection limit of 1 infectious dose that contains 50 to 1,000 virions in picornavirus infection) [3]. The disadvantage is that some expertise and quality control system are required for the cell culture system and for the identification of the cytopathic effect of infected cells. As for the timeliness of reporting, the cell culture-based procedure is time-consuming. It takes for 10 days to confirm the sample as PV-negative even after the introduction of the latest procedure "New Algorism" recommended by WHO [2]. Currently, detection of the circulating vaccine-derived PV (cVDPV) has a high priority in the eradication program and will be in the post-eradication era. Therefore, rapid (at the order of day) and sensitive detection of PV in laboratory diagnosis could contribute to shortening of the timeliness of reporting for mop-up vaccine campaign to control cVDPV outbreaks.

Among currently available procedures detecting RNA viruses, a reverse transcription-loop-mediated isothermal amplification (RT-LAMP) system seems to be a most promising method that meet the demands expected for the cell culture-based isolation procedure [4]. The advantages of RT-LAMP system are; 1) minimum essential equipment is an isothermal heat bath (final results can be visibly observed by the increased turbidity)[5], 2) high sensitivity (detection limits of 0.01 PFU for severe acute respiratory syndrome coronavirus, 0.1 PFU for mumps virus, 0.4 focus forming units for hepatitis A virus, 50 copies of viral genomes for swine vesicular disease virus) [6-9], 3) rapid detection (about 1 h), 4) less possibility of cross-contamination between the samples due to the one-step procedure.

In the present study, we have developed a RT-LAMP system for the detection of enterovirus, including PV. This RT-LAMP system showed a high sensitivity comparable to that of the cell culture system for the detection of PV, HEV-A, and HEV-C, but less sensitivity to HEV-B. This RT-LAMP system would be useful for the direct detection of enterovirus from the stool extracts.

## Results

### RT-LAMP primers for the detection of PV

**Figure 1:**
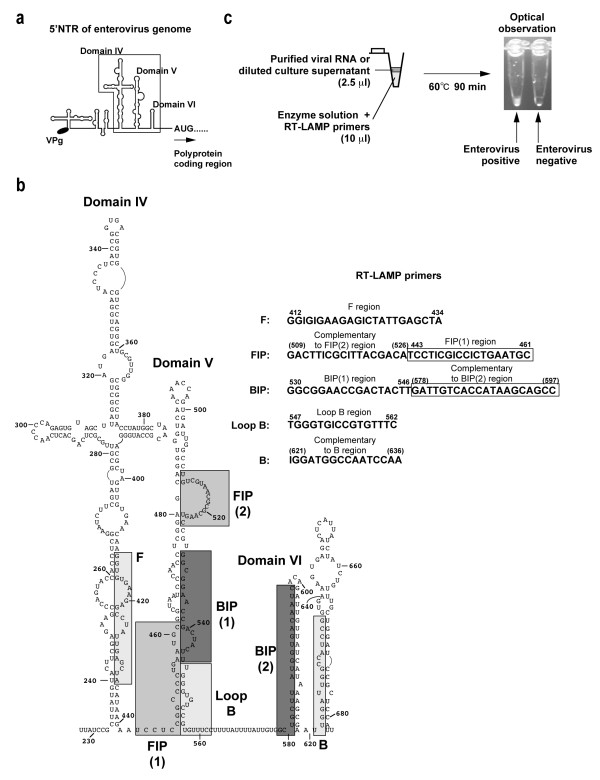
**Regions in the 5'NTR of enterovirus genome examined for the design of RT-LAMP primers**. **a **Schematic view of a model of the secondary structure of 5'NTR of enterovirus genome [22-24]. The region examined for the design of RT-LAMP primers is shown in a box. **b **Primary and secondary structure of 5'NTR of PV1(Mahoney) genome and RT-LAMP primers used in this study. The structure is based on the model proposed by Pilipenko et al. [22]. The region examined for RT-LAMP primers is shown in boxes on the secondary structure. The numbers on the RT-LAMP primers represent corresponding nucleotide positions on the 5'NTR. For primers that have complimentary sequence to the 5'NTR, the numbers are shown in parenthesis. **c **Scheme of RT-LAMP procedure examined in this study.

**Figure 2:**
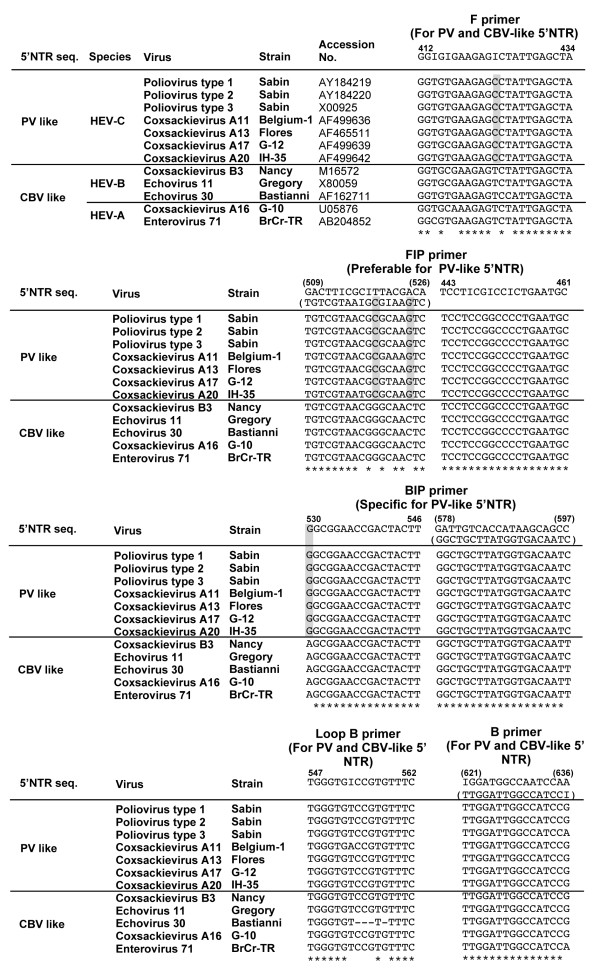
**Comparison of the nucleotide sequences of enterovirus genomes examined for RT-LAMP primers**. Enterovirus genomes are classified into PV-like and CBV-like 5'NTR [10,11]. The nucleotides characteristic to PV-like 5'NTR are highlighted in boxes colored by gray. Primers that have complete match for PV-like 5'NTR near and at the 3' end are presented as preferable and specific primers to PV-like 5'NTR, respectively.

To detect PV by RT-LAMP methods, we analyzed the 5'NTR for the design of the primers (Figure 1a). 5'NTR is known to be classified into two phylogenetic groups based on the primary structure, PV-like or CBV-like 5'NTR [10,11]. PV-like 5'NTR is observed for enteroviruses belonging to *Human enterovirus species C *(HEV-C) and HEV-D, and CBV-like 5'NTR is observed for those belonging to HEV-A and HEV-B, respectively [11]. Therefore, we designed the primer sets to detect PV-like 5'NTR according to conditions required for the primer in RT-LAMP reaction in terms of the location and Tm values of the primers http://loopamp.eiken.co.jp/lamp/primer.html (Figure 1b). Among the 5 primers used in the RT-LAMP reaction, 2 primers were preferable (a complete match for PV-like 5'NTR near the 3' end of the DNA fragment generated in RT-LAMP reaction, FIP primer) or specific (a complete match for PV-like 5'NTR at the 3' end of the DNA fragment generated in RT-LAMP reaction, BIP primer) to PV-like 5'NTR (Figure 2). Other 3 primers (F, B, Loop B primers) were designed with conserved sequences between PV-like and CBV-like 5'NTRs.

### Sensitivity of RT-LAMP system for the detection of PV

**Figure 3:**
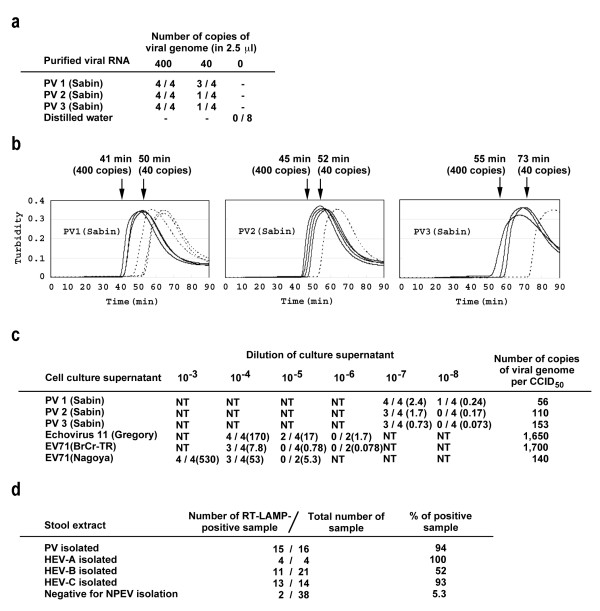
**Sensitivity and specificity of RT-LAMP system**. **a **Sensitivity of RT-LAMP system for purified viral RNA of PV(Sabin) strains. **b **Kinetics of the detection in RT-LAMP system. The average time required for the detection of the signals is shown for each numbers of the copies. **c **Sensitivity and specificity of RT-LAMP system for enterovirus. Cell culture supernatants of the cells infected with enteroviruses were used for the detection of the viral RNA by RT-LAMP system. The numbers in the parenthesis show the titre of virus (CCID_50_) included in the RT-LAMP reactions. The numbers of copies of the viral genome per CCID_50 _are also shown for each virus. NT, not tested. **d **Sensitivity and specificity of RT-LAMP system for the viral RNA purified from stool extracts of AFP cases.

First, the sensitivity of RT-LAMP reaction was examined by using purified viral RNA of PV(Sabin) strains (Figure 3a). In the RT-LAMP reaction, 400 copies of viral genome were detected for all the PV(Sabin) strains (4/4), and 40 copies of viral genome were detected in some samples (1/4 to 3/4). Signals of RT-LAMP were detected within 50 min of the reaction for samples with 400 copies of viral genomes (Figure 3b). For samples with 40 copies of viral genomes, the signals were detected as late as 50 to 73 min.

Next, we examined the sensitivity of RT-LAMP system for enterovirus species by using cell culture supernatant of the virus without viral RNA extraction (Figure 3c). Cell culture supernatant of cells infected with PV(Sabin) strains were RT-LAMP positive at dilution of 1:10,000,000, which contains at least 0.73 to 2.4 CCID_50 _of viruses (about 100 copies of the viral genomes). Echovirus 11 (belonging to HEV-B) and enterovirus 71 (EV71) strains (belonging to HEV-A) showed lower sensitivity in the RT-LAMP reaction compared to PV(Sabin) strains. The detection limit of echovirus 11 and EV71 strains were 17 CCID_50 _(28,000 copies of the viral genome) and 7.8 to 53 CCID_50 _(7,400 to 13,000 copies of the viral genome), respectively.

Finally, we examined to detect PV from the stool samples of AFP cases (Figure 3d). Stool samples that were positive for PV (16 samples), HEV-A (4 samples), -B (21 samples), and -C (14 samples), or negative for enterovirus (38 samples) by cell culture-based isolation were examined. PV and HEV-C were detected with high positive rates in the stool samples by RT-LAMP (94 and 93%, respectively). Unexpectedly, HEV-A was also detected with a high positive rate (100%), and HEV-B was also detected with a relatively high positive rate (52%). For stool samples negative for enterovirus, 2 samples (derived from one AFP case) were positive by RT-LAMP. For these samples, enterovirus 96 was identified by sequence analysis of VP1 coding region utilizing a seminested PCR [12] (data not shown). Therefore, this RT-LAMP system showed a good correlation with the cell culture-based isolation especially for PV, HEV-C, and HEV-A.

**Figure 4:**
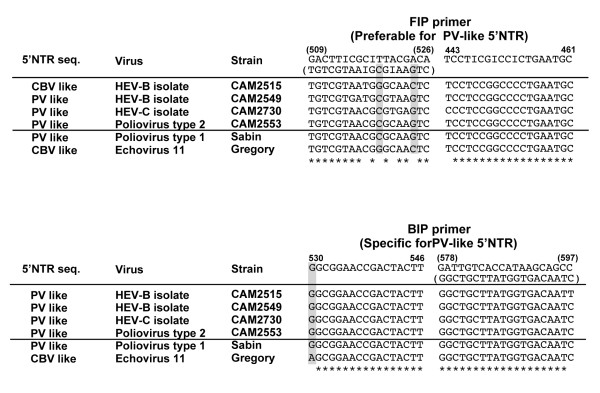
**Comparison of the nucleotide sequences of the 5'NTR in the viral genomes of enterovirus isolates**. The nucleotides characteristic to PV-like 5'NTR are highlighted in boxes colored by gray.

We analyzed the sequence of HEV-B isolates (CAM2515 and CAM2549) that were positive for RT-LAMP, and for a HEV-C (CAM2730) and a PV2 (CAM2553) strains that were negative for RT-LAMP (Figure 4). The 5'NTR of these HEV-B isolates showed mixed genetic properties of PV-like 5'NTR and CBV-like 5'NTR. The 5'NTR of CAM2515 showed CBV-like sequence in the FIP primer-binding region, but has a PV-like sequence in the region for BIP primer. The 5'NTR of another HEV-B isolate CAM2549 and a HEV-C isolate CAM2730 showed similar sequence to PV-like 5'NTR in these regions. The sequence of the 5'NTR of PV2 isolate CAM2553 was similar to its parental PV2(Sabin).

## Discussion

In this study, we have developed a RT-LAMP system for a rapid and highly sensitive detection of enterovirus including PV directly from stool samples of AFP cases without cell culture-based procedures. 5'NTR of enteroviruses is classified into two groups based on its primary structure, PV-like or CBV-like 5'NTR [10,11]. Actually, a RFLP assay utilizing *Bst*OI have been developed to differentiate these genogroups [13]. We designed RT-LAMP primers preferably to detect PV-like 5'NTR rather than PV-specific primers, because we could not find nucleotide sequences specific to PV strains but not to other HEV-C strains in the 5'NTR. Actually, cVDPVs with unknown nucleotide sequences in the 5'NTR, which was probably derived from the viral genome of other non-polio enterovirus, were isolated [14].

**Figure 5:**
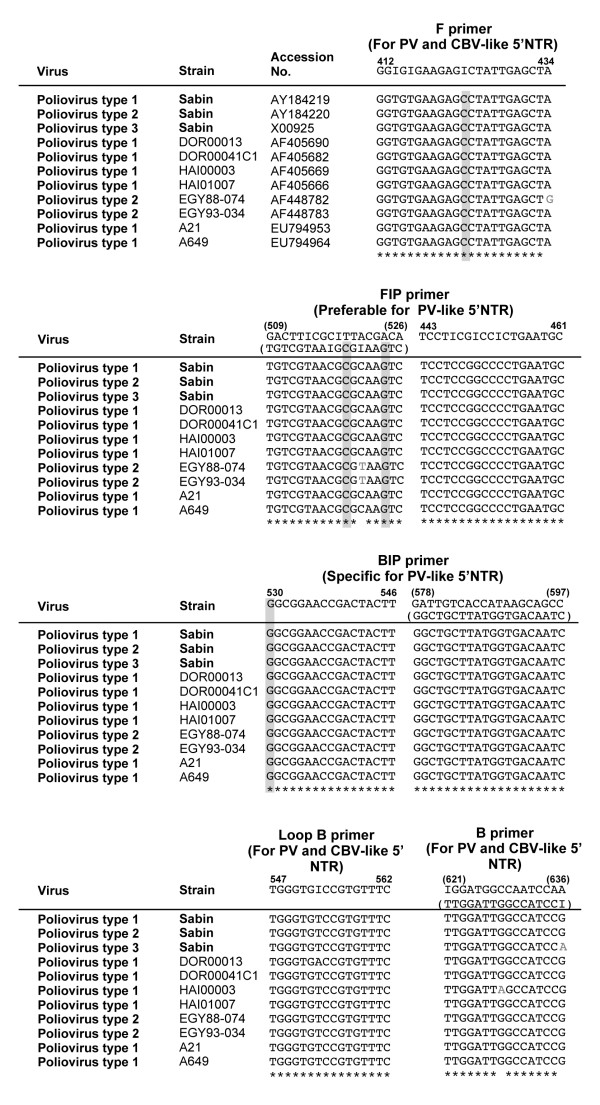
**Comparison of the nucleotide sequences of the regions in the viral genomes of cVDPV and iVDPV strains examined for RT-LAMP primers**. The nucleotides characteristic to PV-like 5'NTR are highlighted in boxes colored by gray.

In the primers examined in this study, the specificity to PV-like 5'NTR was defined by 2 primers (FIP and BIP primers, Figure 2). The sequences of primers we used might detect most of the cVDPVs or immunodeficient VDPVs, which were circulating or infecting for about 2 to 10 years [14-16] (Figure 5). However, one cVDPV strain (EGY88-074), which was isolated in an early stage of the circulation in Egypt with its 5'NTR that was probably derived from other enterovirus genomes by recombination [14], contained a different nucleotide at the 3' end of F primer. This nucleotide change was not observed for another cVDPV strain (EGY93-034), which was isolated in the late stage of the circulation, suggesting that this nucleotide change was not stable during the circulation. Therefore, some cVDPV isolates with this rare mutation might not be detected with the primer set examined in this study.

We observed the specificity to PV-like 5'NTR to some extent compared to CBV-like 5'NTR in the RT-LAMP reaction, where about 100-fold difference was observed in the sensitivity (Figure 3c). However, detection of enteroviruses from stool samples showed only slightly lower positive rates for HEV-A and HEV-B (100 and 52%, respectively) compared to those for PV and other HEV-C (94 and 93%, respectively) (Figure 3d). The relatively low positive rates of HEV-B among these virus species might depend on the designed specificity of RT-LAMP to PV-like 5'NTR. Sequence analysis of 5'NTR of HEV-B isolates indicated that these field isolates could have some genetic features similar to PV-like 5'NTR (Figure 4). Therefore, with relatively low specificity of RT-LAMP system (100-fold difference between PV-like and CBV-like 5'NTR), the mixed genetic features of the 5'NTR of HEV-B field isolates might have affected the specificity of the RT-LAMP system.

An essential factor of the sensitivity of RT-LAMP system for the detection of PV from stool extracts seems to be the amount of viral RNA available for the reaction in addition to the quality of the RNA. We found one stool sample (CAM2553) was negative by the RT-LAMP among the 16 stool extract that was positive for PV by cell culture (Figure 3d). The 5' NTR of the PV2(CAM2553) did not have any nucleotide changes from that of its parental PV2(Sabin) strain (Figure 4). It is plausible that the low amount of viral RNA in the sample caused this false-negative result under the detection limit of RT-LAMP. The amounts of PV in the stool extracts were not generally high (< 10^0.5 ^to 10^2.5 ^CCID_50_/50 μl, Table 1). In the RT-LAMP reaction examined in this study, we purified viral RNA from 200 μl of stool extract and collected in 50 μl of elusion buffer (4-fold concentration by this procedure), and then 2.5 μl of this purified viral RNA solution was used for RT-LAMP reaction. Therefore, the net amount of viral RNA used in RT-LAMP reaction corresponds to that contained in 10 μl of stool extract. For the isolation of PV, 200 μl of stool extract is inoculated into the cells. Therefore, there is 20-fold difference in the available viral RNA or the infectious units between the RT-LAMP system and the cell culture system because of the intrinsic difference of the scale of the assay (12.5 μl vs. 1.0 ml). Because of a high particle-to-infective-unit ratio of PV, which was estimated as 56 to 153 copies of viral genome per CCID_50 _in this study (Figure 3c), relatively high sensitivity was attained in RT-LAMP system almost comparable to that of cell culture-based isolation. It should be noted that the sensitivity of this RT-LAMP system (< 400 copies) was not high compared to those of optimized RT-LAMP systems (1-100 copies), and was lower than that of a conventional real-time PCR system [17] (< 10 copies). Additional procedures to increase the concentration of viral RNA and to improve the quality of RNA would be helpful to improve the sensitivity of the RT-LAMP system for the detection of PV from stool extracts.

**Table 1:** Titre of PV in stool samples

Stool sample	Isolated PV	Virus titre (CCID_50_/50 μl)
CAM2553	PV2	< 10^0.5^
CAM2554	PV1+2	< 10^0.5^
CAM2885	PV3+NPEV	< 10^0.5^
CAM2896	PV2	10^1.5^
CAM2897	PV2	10^1.75^
CAM2906	PV3	10^0.75^
CAM2907	PV3	10^0.75^
CAM2936	PV3+NPEV	< 10^0.5^
CAM2937	PV3+NPEV	< 10^0.5^
CAM2970	PV1+PV3	10^0.75^
CAM2995	PV3+NPEV	< 10^0.5^
CAM2996	PV3	10^2.5^
CAM3017	PV2	< 10^0.5^
CAM3018	PV2	10^1.5^
CAM3044	PV2	< 10^0.5^
CAM3045	PV2	10^1.5^

## Conclusions

In summary, we developed a highly sensitive RT-LAMP system for the detection of enterovirus, including PV, from the stool extracts. The cell culture-based isolation will be needed for genetic characterization of PV isolates, particularly differentiation of wild, VDPV, and mixtures of PV and enterovirus. The RT-LAMP system would be useful for a triage of overwhelming number of clinical samples to reduce the workload and to minimize the timeliness of the report by identifying the samples negative for PV within a day.

## Methods

### Cells, viruses, and clinical samples

RD cells (human rhabdomyosarcoma cell line) were cultured as monolayers in Dulbecco's modified Eagle medium (DMEM) supplemented with 10% fetal calf serum (FCS) and used for titration of viruses. Virus titre was determined by measuring 50% cell culture infectious dose (CCID_50_) at 35°C by a microtitration assay [18]. Stool extracts from AFP cases were used for isolation of enterovirus and for RT-LAMP reaction. The species and serotypes of enterovirus isolates were determined by sequencing of the viral genome with a primer set for 2BC coding region (2A2+ and 2C-primers) [19] and with those for VP1 coding region (292 and 222 primers) [20]. All the clinical samples and virus isolates used in this study are appropriately anonymized. Therefore, they are exempt from the regulation under the Committee for Ethical Regulation of the National Institute of Infectious Diseases.

### RNA purification

Viral genomic RNA was purified from the stool extracts of AFP cases by using a High Pure viral RNA purification kit (Roche). In this purification procedure, viral genomic RNA was collected in 50 μl of distilled water purified from 200 μl of stool extracts.

### RT-LAMP reaction

Primers used in this study are shown in Figure 1 (Figure 1b). Stocks of the primers were prepared in distilled water in concentrations as follows; 40 μM for FIP and BIP primers, 5 μM for F and B primers, and 20 μM for Loop B primers. RT-LAMP reaction was performed by using a RNA Amplification Kit (RT-LAMP) (Eiken Chemical Co. Ltd., Tokyo, Japan). RT-LAMP reaction was prepared according to the manufacturer's instruction but in a total 12.5 μl reaction. The final concentrations of the primers were as follows; 1.6 μM for FIP and BIP primers, 0.2 μM for F and B primers, and 0.8 μM for Loop B primers. In the total 12.5 μl reaction, 2.5 μl of purified viral RNA solution or diluted cell culture supernatant were included (Figure 1c). RT-LAMP reaction was performed at 60°C for 90 min and optical density at 650 nm was measured as the turbidity by a Loopamp Realtime Turbidimeter LA-320C (Teramecs, Kyoto, Japan). The threshold of the turbidity for RT-LAMP positive sample was defined at 0.1 in the measurement [5]. The numbers of copies of the viral RNA of PV Sabin strains and prototype enterovirus strains were determined by real-time TaqMan PCR system developed by Nijhuis et al. [17], as described previously [21].

## Competing interests

The authors declare that they have no competing interests.

## Authors' contributions

MA carried out the development of RT-LAMP system for enterovirus. HL and DY carried out the molecular genetic analysis of enterovirus isolates. MA, YN, HY, HS carried out the isolation of enteroviruses. MA planned the project and designed experiments. MA and HS wrote the manuscript. TW and HS supervised the laboratory works. All authors read and approved the final manuscript.

## Pre-publication history

The pre-publication history for this paper can be accessed here:

http://www.biomedcentral.com/1471-2334/9/208/prepub
